# Exploring the Experiences and Perspectives of Patients With Early Breast Cancer, Caregivers, and Health Care Professionals: Italian Social Media Listening Study

**DOI:** 10.2196/73371

**Published:** 2026-03-24

**Authors:** Matteo Basilio Suter, Rosanna D'Antona, Manuelita Mazza, Enrica Francavilla, Alice Messi, Abhinav Maurya, Shiva Padhi, Diletta Valsecchi

**Affiliations:** 1 Novartis Farma S.p.A., Viale Luigi Sturzo, 43 - 20154, Milan, Italy, 39 3442549217; 2 Europa Donna Italia, Milan, Italy; 3 Cancer Institute Fatima bint Mubarak Center - Cleveland Clinic Abu Dhabi, Abu Dhabi, United Arab Emirates; 4 Novartis Healthcare Pvt. Ltd., Hyderabad, India

**Keywords:** early breast cancer experiences, social media listening, social media analysis, patient journey mapping, quality of life in cancer, diagnosis and treatment challenges, health care professional, patient-HCP communication, emotional and physical well-being, unmet needs in cancer care, breast cancer recurrence

## Abstract

**Background:**

Published evidence on patient experiences, perceptions, and challenges related to early breast cancer (eBC) in Italy is limited. Understanding these aspects is critical for improving diagnosis, treatment outcomes, and quality of life (QoL).

**Objective:**

This study used social media listening (SML) to explore the patient journey, treatment perceptions, QoL, and unmet needs of patients with eBC, caregivers, and health care professionals (HCPs) in Italy.

**Methods:**

This retrospective noninterventional SML study analyzed publicly available posts from December 2021 to November 2023 using breast cancer–related keywords in English and Italian through Sprinklr, a web-based aggregator tool. Posts sourced from social media platforms, such as X (formerly known as Twitter), blogs, forums, Facebook, Instagram, and YouTube, were filtered by geographic location to include only users in Italy. Posts were filtered using natural language processing (NLP) for relevance and duplicates, followed by manual review and stakeholder identification (patients, caregivers, and HCPs). Key themes of discussion were identified through thematic analysis of posts across the stages of the patient journey (symptoms, diagnosis, treatment, etc). Ethical guidelines were followed by using anonymized, publicly available data. Descriptive statistics were used to analyze the data, and posts with missing data were excluded. Consequently, denominators varied across analyses and were adjusted based on data availability for specific variables.

**Results:**

Of the 20,008 posts initially extracted, 1580 posts were retained following NLP filtering, and 530 posts were included after manual screening. The majority (493/518, 95%) of the posts were sharing information about diagnosis and treatment journeys, emotional challenges, QoL concerns, and symptoms (eg, lumps, breast pain), while 27% (141/518) of the posts sought information on diagnostic dilemmas, treatment options, and second opinions. Patients contributed 60% (318/530) of the posts, and caregivers contributed 21% (111/530) of the posts, with over half (57/107, 53%) discussing their mothers’ diagnosis and treatment struggles. HCPs contributed 16% (85/530) of the posts, primarily sharing clinical trial updates, drug approvals, and disease awareness efforts. A total of 88 posts included discussions on QoL, and eBC significantly impacted patients’ emotional, physical, functional, and social well-being. Discussions revealed key unmet needs, including limited awareness of adjuvant therapy options, lack of peer support groups, suboptimal patient-HCP communication, and insufficient access to specialty care facilities.

**Conclusions:**

This study highlights gaps in eBC management related to patient education, HCP communication, and access to specialty care and describes an associated worsening of QoL for patients as reflected in social media posts. Within the limitations of an observational SML design, increasing patient and caregiver awareness of available adjuvant therapies to improve adherence and reduce recurrence risk, alongside expanding access to regional breast cancer centers, may help optimize patient experiences and outcomes. Further research using complementary data sources is needed to confirm and extend these findings.

## Introduction

In the year 2022, female breast cancer (BC) was projected to be the most prevalent form of cancer globally, accounting for 2.3 million new cases, which constitutes 11.6% of all cancer diagnoses [1]. It is also the leading cause of death among women, accounting for 670,000 deaths globally, and ranked fourth in terms of cancer-related mortality worldwide [1]. Similarly, BC is the most commonly diagnosed cancer among women in Italy. It was estimated that there would be a total of 55,900 new cases in 2023, with over 90% of them being detected in the early stages [2,3].

Despite the availability of various treatment options for early BC (eBC), a significant number of patients still experience recurrence. For luminal BC, there is a long-term cumulative risk, with Italy reporting up to 10.5% distant recurrence within the first 5 years [4].

Patients with BC encounter numerous challenges throughout their journey, from identifying symptoms to receiving a diagnosis, undergoing treatment, and managing the disease [5-7]. Early diagnosis and effective management of BC are crucial in improving patient outcomes and reducing health care expenses [8,9]. To understand the diverse challenges of patients with eBC, conventional methods, such as questionnaires, interviews, and patient-reported outcome measures, are often used to gather qualitative data. However, these studies frequently involve a limited number of participants or require extended durations to be conducted, resulting in a limited understanding of the unique obstacles faced by individual patients [10-13]. Recently, social media listening (SML) has gained prominence as an approach to gain deeper insights into these challenges [14-16].

High internet penetration and presence of various social media platforms have greatly influenced health care [17,18]. Social media platforms have become valuable sources of information, allowing patients to share their beliefs about diseases, treatment experiences, satisfaction with outcomes, and other factors that impact their lives [19].

SML is a new approach for gathering information from social media platforms and can be useful in generating insights from user experiences. SML has been used to monitor discussions on health-related topics in various cancers [16,19-25]. However, there is limited published literature on the use of SML to investigate the needs and experiences of patients with eBC [15]. This study explores SML as a research tool to provide insights into the journey of patients with eBC.

This study aimed to understand the patient journey, treatment perceptions, quality of life (QoL), unmet needs, and concerns of patients with eBC by analyzing the social media posts from patients, caregivers, and health care professionals (HCPs).

## Methods

### Study Design and Data Source

This study was a noninterventional retrospective analysis of social media data available in the public domain. A comprehensive search was conducted on various social media platforms, including X (formerly known as Twitter), blogs, forums, Facebook community groups, Instagram, and YouTube, from December 2021 to November 2023 using keywords related to BC in both Italian and English languages. The initial keyword list was developed by a multidisciplinary study team comprising researchers with expertise in oncology, social media analytics, and qualitative research. The team first compiled candidate terms based on clinical terminology (eg, “early breast cancer,” “chemotherapy”) and commonly used lay terms (eg, “chemo,” “cancer journey,” “tumor,” “lump”), informed by prior SML and patient-experience literature [19,26]. Preliminary scoping searches were then conducted in the Sprinklr tool to test and refine this list; identify additional relevant terms and hashtags used by patients, caregivers, and HCPs; and optimize precision and recall. Boolean operators (eg, AND, OR) were used to structure the search strings, and all terms were translated into both Italian and English to reflect the bilingual nature of online discourse in Italy. This iterative, expert-informed process aligns with best practices in SML research [19,27,28] and was used to finalize the search strategy prior to full data extraction. The data have been reported in accordance with the SRQR (Standards for Reporting Qualitative Research) checklist (Checklist 1). The specific search terms and list of forums and blogs are reported in Multimedia Appendix 1.

Each social media platform was selected based on its potential to capture unique dimensions of the patient, caregiver, and HCP experience. The inclusion of multiple platforms allowed this study to triangulate insights from different types of user engagement:

- X: Real-time updates, public sentiment, and awareness campaigns
- Facebook groups: Peer support, community discussions, and shared experiences
- Forums: Anonymous, in-depth discussions among patients and caregivers
- Blogs: Detailed personal narratives and reflections on the cancer journey
- Instagram and YouTube: Visual storytelling and video-based sharing of experiences

While blogs are not inherently discussion-based, they offer rich, first-person accounts of the lived experience of illness. Prior SML studies have consistently included blogs as valuable sources of qualitative data [19,26,27].

Forums and blogs were identified through Sprinklr’s indexing of publicly accessible websites and were selected if they (1) hosted user-generated content related to BC, (2) were accessible without registration or password protection, and (3) had contributors whose profiles or content indicated an Italian context (eg, language, self-reported location).

### Eligibility Criteria

This study included posts that (1) were publicly available (ie, not private, password-protected, or restricted content); (2) originated from users located in Italy (based on profile or geolocation information); (3) contained 1 or more keywords related to BC; and (4) appeared to reflect the experiences, perceptions, questions, or opinions of patients with eBC, their caregivers, or HCPs involved in BC care. This study excluded (1) posts that were clearly unrelated to BC, despite containing 1 or more keywords (eg, spam, advertisements, financial content); (2) posts from news portals, media outlets, and scientific journals; (3) posts with commercial or promotional content; and (4) duplicate post links. These eligibility criteria were applied consistently across all platforms and are reported in detail in Multimedia Appendix 1.

### Data Collection, Selection of Posts, and Data Cleaning

Data from public posts were collected using Sprinklr [29], a web-based aggregator tool. This tool allows downloading of posts along with the date stamp and geographic location information of the users. Based on the geographic location of the user profile, only posts from Italy were downloaded. Each post was assigned a unique ID by the tool. Data filtration techniques were applied using exclusion keywords to remove junk data to maintain data quality using natural language processing (NLP). Furthermore, NLP was used to clean the data and eliminate duplicates for reliable analysis.

### Data Analysis

**Figure 1.**
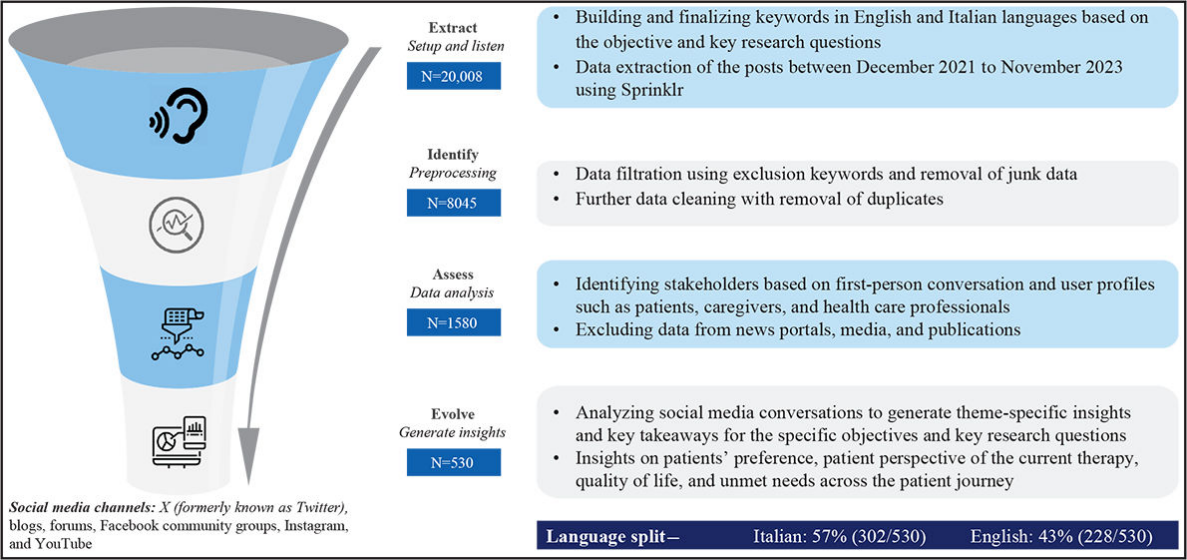
Social media listening (SML) methodology: from extraction of posts to insights generation. “N” denotes number of posts.

Manual screening was conducted to analyze the data. Each post was indexed to identify the stakeholders based on first-person conversations and user profiles, including patients, caregivers, and HCPs. Posts related to news portals, media, and publications were excluded to focus solely on social media posts from patients, caregivers, and HCPs. The analysis included examining the posts based on the social media channel (X, blogs, forums, Facebook community groups, Instagram, and YouTube), the tone of the posts (positive, neutral, or negative), and the key themes of discussion (Figure 1). The themes were identified based on the type of information relevant to the study objective discussed in the posts. A single post could be categorized under more than 1 theme based on the information mentioned in the post.

The thematic analysis in this study followed a predominantly deductive approach. The themes were derived based on a conceptual framework aligned with the typical patient journey for individuals diagnosed with eBC. This framework included key stages such as symptom onset, diagnosis, treatment, disease management, and quality of life. Additionally, this study incorporated themes related to online patient behavior, such as sharing disease awareness and discussing clinical trial or research updates, which are commonly observed in social media discourse among patients, caregivers, and HCPs.

The deductive nature of the thematic analysis was guided by predefined categories informed by existing literature on the eBC patient journey and prior SML studies [19,28]. These categories were used to structure the coding process and ensure alignment with the study objectives. While the primary approach was deductive, the analysis remained open to emergent subthemes within each category during manual screening, allowing for flexibility in capturing nuanced insights. Each post was coded and analyzed by a qualified researcher with expertise in oncology, social media analytics, and qualitative research.

The theoretical underpinning of this approach draws from the patient-centered care model and the concept of the illness trajectory, which emphasizes understanding the lived experiences of patients across different stages of their condition. This framework supports the identification of themes that reflect both clinical milestones and psychosocial dimensions of the patient experience. The review and coding were led by a primary analyst (A Maurya), who has expertise in oncology, social media analytics, and qualitative research, using structured spreadsheets and a predefined codebook. To enhance the reliability of the reviewing and coding process, we applied techniques, such as iterative codebook refinement and regular team discussions, to review preliminary themes and ensure consistency with the study objectives. Data analysis and interpretation were led by A Maurya in collaboration with SP, with input from the other coauthors, who contributed to the refinement of themes and final interpretation of the findings.

For analysis and reporting, the terms “post” and “mention” were used. The term “post” referred to a single post. The term “mention” referred to the number of times a symptom, treatment, diagnostic test, or other parameter was mentioned in each post. It is important to note that the number of mentions was independent of the number of posts. The unit of analysis is posts and mentions, not unique individuals, and due to data anonymization, users could not be tracked across different platforms. Descriptive statistics were used to analyze the data, which were presented in the form of post counts (N), mention counts (n), and percentages (%). The percentage calculation was performed by dividing the number of posts or mentions by the total number of posts or mentions providing specific information, respectively. Missing data were neither imputed nor considered in the analysis. Consequently, denominators varied across analysis and were adjusted based on the availability of data for specific variables.

### Ethical Considerations

All data used in this SML study were collected exclusively from publicly accessible sources, without accessing any password-protected or nonpublic information. This study involved social media analysis and did not report any data with identifiable participants. All web-based content was anonymized and is in compliance with the Health Insurance Portability and Accountability Act (HIPAA). Approval was obtained from the Novartis safety registry 1P1R—the governing body that holds oversight on the use of social media by Novartis (DE014963-V1). All relevant local and global laws affecting and relating to the use of social media, aligned with and as reflected in Novartis processes, were followed in the conduct of this study. The authors of this study consent to the publication of the submitted manuscript and declare that no individual patient data requiring consent have been presented.

## Results

### Overview

A total of 20,008 posts were initially extracted from social media platforms using BC-related keywords during the study period. After filtration using exclusion keywords, removal of junk data and further data cleaning with the removal of duplicates using NLP, it resulted in retaining 1580 posts. These 1580 posts were then screened manually to check for relevance and identify stakeholders based on first-person conversation and user profiles, such as patients, caregivers, and HCPs. After excluding nonrelevant data, such as data from news portals, media, and publications, a total of 530 posts were included in the final analysis for insights generation (Figure 1).

Most posts were from patients (318/530, 60%), compared with 21% (111/530) from caregivers; 16% (85/530) from HCPs; and 3% (16/530) from unidentified persons. Of the 448 posts mentioning the cancer type, 95% (427/448) mentioned de novo BC and 5% (21/448) mentioned recurrent BC. Of the 107 posts mentioning the caregiver relationship with the patient, 53% (57/107) were posts from children discussing their mothers’ diagnosis and treatment challenges. Patients’ current age was reported in 89 posts, with most (56/89, 63%) posts reporting the patient age range of 21‐50 years. Time since diagnosis was reported in 90 posts, with 31% (28/90) of the posts mentioning patients being diagnosed within 6 months to 1 year. Demographic and clinical characteristic details are reported in Multimedia Appendix 2.

### Social Media Landscape and Online Behavior

#### Social Media Platform Distribution and Interaction Trends

Forums and X were the most commonly used social media platforms, accounting for 47% (249/530) and 31% (164/530) of the posts respectively, followed by Facebook (69/530, 13%), YouTube (21/530, 4%), Instagram (16/530, 3%), and blogs (11/530, 2%). Posts from 10 forums and 5 blogs were included in the analysis, and a list of forums and blogs is available in Multimedia Appendix 1. Among the forums, Medicitalia^+^, Aimac, Triste Mondo, and Carenity were the forums with the most posts. Most posts concentrated on the treatment and management of the disease, followed by discussions on diagnosis (Table 1). Among the 518 posts categorized as either sharing or seeking information, approximately 95% (493/518) involved sharing information, while only 27% (141/518) sought information.

**Table 1.** Discussion themes from the posts.

Topic of conversation	Mentions of the specific topic of conversation from the total mentions (n=1037), n (%)
Treatment and management	425 (41)
Diagnosis	249 (24)
Quality of life	135 (13)
Symptoms	124 (12)
Disease awareness	73 (7)
Clinical trial or research update	31 (3)

#### Sharing Information

Patients shared various aspects of their experiences, providing insights into their diagnostic journey, emotional support needs, and treatment courses. In discussing their diagnostic journey, patients shared their experiences of undergoing multiple diagnostic tests and the associated waiting periods, aiming to reassure their peers who were facing similar challenges. Time since diagnosis was reported in 90 posts. Regarding emotional support, 17% (15/90) of the posts mentioning a recent diagnosis frequently expressed anxiety and concern about the progression of their treatments, whereas 12% (11/90) of the posts mentioning a diagnosis 4 to 6 years earlier were particularly active in addressing queries related to available treatment options and medication choices. Concerns about HCP interactions were also prevalent, with some patients expressing dissatisfaction due to multiple visits and the perceived lack of cordiality from certain HCPs. Furthermore, patients detailed the impact of their condition on their overall QoL and described pain, discomfort, and the effects on personal relationships and overall well-being.

#### Seeking Information

In the context of information-seeking behavior, patients expressed various concerns, including the interpretation of ambiguous symptoms, wherein they sought clarification from peers regarding concerning lumps or masses, as online resources often exacerbated confusion and anxiety. Additionally, there were diagnostic dilemmas, with individuals inquiring about preparation for upcoming breast examinations and biopsy results. Regarding treatment options, patients asked about improved disease management strategies to overcome the adverse effects of medications. Furthermore, patients expressed apprehension about medical consultations, especially regarding the frequency of medical consultations in the absence of clear guidance, and frequently sought second opinions from HCPs. This was largely driven by anxiety and worry induced by online testimonials read by patients.

A summary of insights from individual social media channels is provided in Multimedia Appendix 3.

### Insights From Patients’ and Caregivers’ Discussions

#### Online Engagement and Support Seeking Pattern

Insights from patients’ and caregivers’ discussions highlighted that women aged 31‐40 years, as reported in 26% (23/89) of the posts, were more active in sharing their patient journey and seeking peer experiences and community inclusiveness. Online platforms served as their preferred method for seeking clarification and addressing concerns, with approximately 30% (28/90) of the posts mentioning active online engagement within the first year of their diagnosis.

#### Symptoms

Key symptoms reported by patients included lumps, cysts, and breast pain (Table 2). Of the 128 mentions on symptoms, approximately 33% (42/128) of the mentions revealed that women identified a lump or mass as the first symptom through self-examination after a diagnostic checkup. Some mentions revealed that patients discovered their BC only through routine mammograms, as they were asymptomatic. Individuals educated themselves on self-examination at home by reading patient testimonials or watching videos or blogs from peers or medical websites. However, the uncertainty surrounding initial symptoms left patients unsure about whether to wait or visit a doctor, as the information available online was inconclusive. The physical impact of the symptoms, such as changes in breast appearance and severity of nipple discharge, made patients feel self-conscious and uncomfortable.

**Table 2.** Key symptoms reported in posts.

Key symptom	Mentions (%) of the specific symptom from the total mentions (n=128), n (%)
Lumps or mass	42 (33)
Cyst or calcification	20 (16)
Breast pain	17 (13)
Skin irritation	15 (12)
Nodules	14 (11)
Swelling of breasts	12 (9)
Nipple discharge	5 (4)
Others	5 (4)

#### Diagnosis

##### Diagnosis and Monitoring: Procedures, Preferences, and Concerns

**Figure 2.**
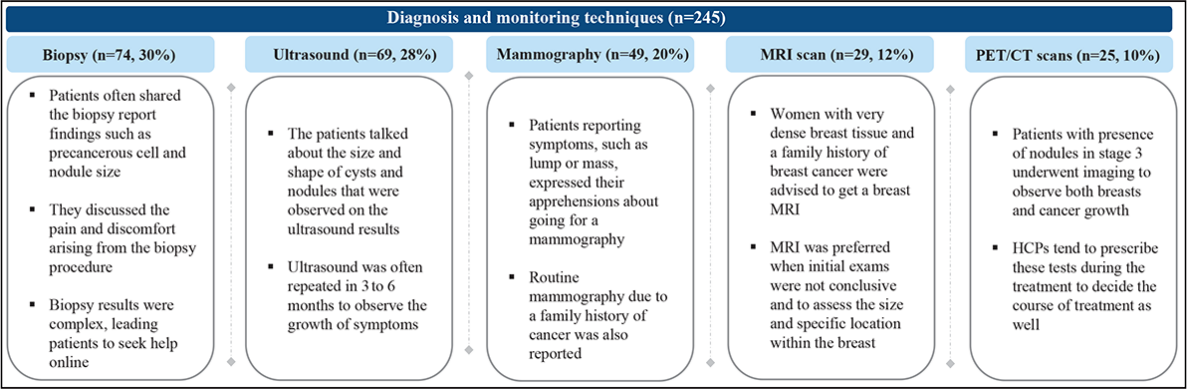
Diagnosis and monitoring techniques reported in posts. “n” denotes the number of mentions. CT: computed tomography; HCP: health care professionals; MRI: magnetic resonance imaging; PET: positron emission tomography.

Diagnostic procedures, such as biopsy, ultrasonography, and mammography, played a vital role in confirming the diagnosis, accounting for 77% (190/245) of the mentions. These procedures were often repeated to establish a definitive diagnosis by radiologists and oncologists (Figure 2).

The analysis of diagnostic and monitoring techniques from 245 mentions highlighted distinct preferences and concerns among patients. Biopsy, representing 30% (74/245) of the mentions, was frequently discussed, with patients sharing results that revealed conditions, such as precancerous cells and nodule sizes. Of the 73 mentions in which patients underwent biopsy, 22% (16/73) reported experiencing pain and discomfort after the procedure and often sought additional online resources due to the complexity of the biopsy results. Ultrasound, accounting for 28% (69/245) of the mentions, frequently involved discussions about the size and shape of cysts and nodules observed in the results, with follow-up ultrasounds typically scheduled every 3 to 6 months to monitor symptom progression. Mammography, mentioned in 20% (49/245) of the mentions, commonly induced patient apprehensions, especially in those reporting symptoms, such as lumps or masses. It was also routinely used by individuals with a family history of cancer. Magnetic resonance imaging (MRI) scans, comprising 12% (29/245) of the mentions, were particularly useful for patients with dense breast tissue and a significant family history of BC. MRIs were preferred when initial exams lacked conclusive results, offering a comprehensive assessment of the size and specific locations of the tumors within the breast. Positron emission tomography (PET) or computed tomography (CT) scans, constituting 10% (25/245) of mentions, were predominantly used to evaluate nodule presence in advanced cancer stages and track cancer growth. These scans were crucial during treatment phases to assist HCPs in determining appropriate treatment courses. Overall, these findings underscored the diverse diagnostic approaches and patient experiences in breast health monitoring.

##### Key Drivers for Diagnosis

Drivers for diagnosis included routine mammography and ultrasound screening, particularly among younger adults who were more informed. A family history of cancer made patients more vigilant in noticing symptoms and undergoing regular screenings. The self-examination for BC symptoms, proactive communication from gynecologists or breast specialists, and clear guidance from oncologists were also key factors.

### Treatment

#### Surgery to Adjuvant Therapies: Experiences and Expectations

Before initiating treatment, patients often sought validation online for their prescribed treatments. Mastectomy was the most mentioned surgical method (137/232 mentions, 59%) for managing local cancerous cells at the first stage. After surgery, oncologists advised patients to undergo histological analysis to determine the further treatment course. Specific drug names were rarely discussed, but patients sought medications that had fewer side effects and higher efficacy. Some common chemotherapy and hormonal drugs mentioned for the first to third steps of treatment included paclitaxel, letrozole, and tamoxifen among others. Patients expressed anxiety about starting adjuvant therapies due to concerns about severe side effects. Adjuvant therapy was used intermittently to provide better relief to patients at high risk of disease progression. From the patients’ and caregivers’ perspective, HCPs rarely had transparent conversations with patients about the risks associated with hormonal or adjuvant therapies.

#### Treatment Sentiment

**Figure 3.**
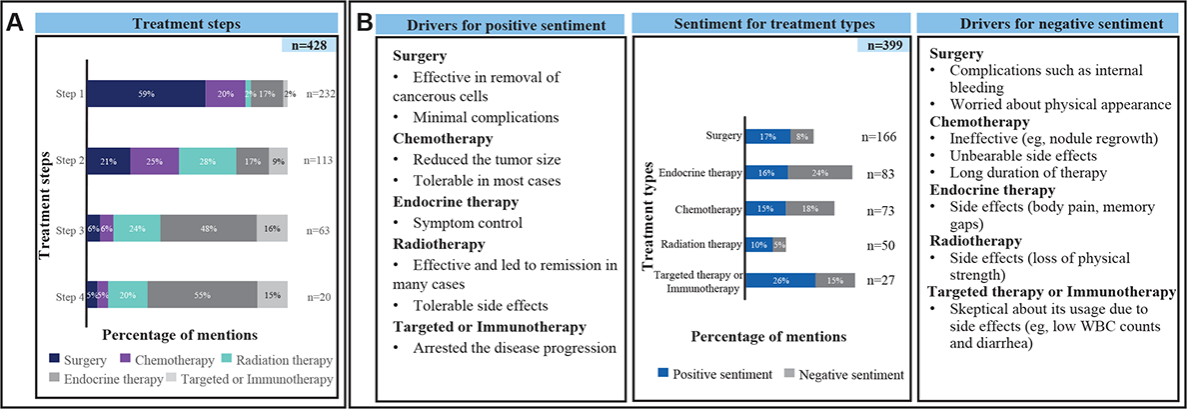
(A) Treatment steps and (B) treatment sentiment. “n” denotes the number of mentions. WBC: white blood cell.

Patients experienced relief from surgery due to effective cancer cell removal and minimal complications. However, long-term treatments were challenging, negatively affecting their QoL. Chemotherapy reduced tumor size but had severe side effects and long durations. Endocrine therapy helped with symptom control but caused issues, such as body pain and memory gaps. Radiation therapy was effective, leading to remission but also resulted in the loss of physical strength. Targeted therapy or immunotherapy arrested disease progression but was met with skepticism due to side effects, such as low white blood cell counts and diarrhea (Figure 3).

### Unmet Treatment Needs

The treatment journey for patients with eBC is often further complicated by a lack of therapy knowledge and poor patient-HCP interactions. One of the key unmet needs identified is the perception of care.

- Limited treatment knowledge: Patients frequently lack awareness of available adjuvant treatment options that are critical for effective eBC management.
- Lack of trusted online peer groups: During treatment initiation, patients often seek support from trusted online peer groups, authenticated Google searches, or medical blogs. However, the absence of a standardized care system leaves them without proper guidance during this challenging period.
- Limited access to specialty breast care units: There is a pressing need for more tertiary cancer care centers across various regions to enhance patient access to specialized care.
- Poor patient-HCP communication: Treatment steps are often not openly discussed by HCPs during consultations, which places the burden of understanding and managing the treatment course on the patients themselves.
- Need for advanced drugs: Patients with recurrence highlighted the need for more advanced drugs to prevent recurrence.

### Quality of Life

**Figure 4.**
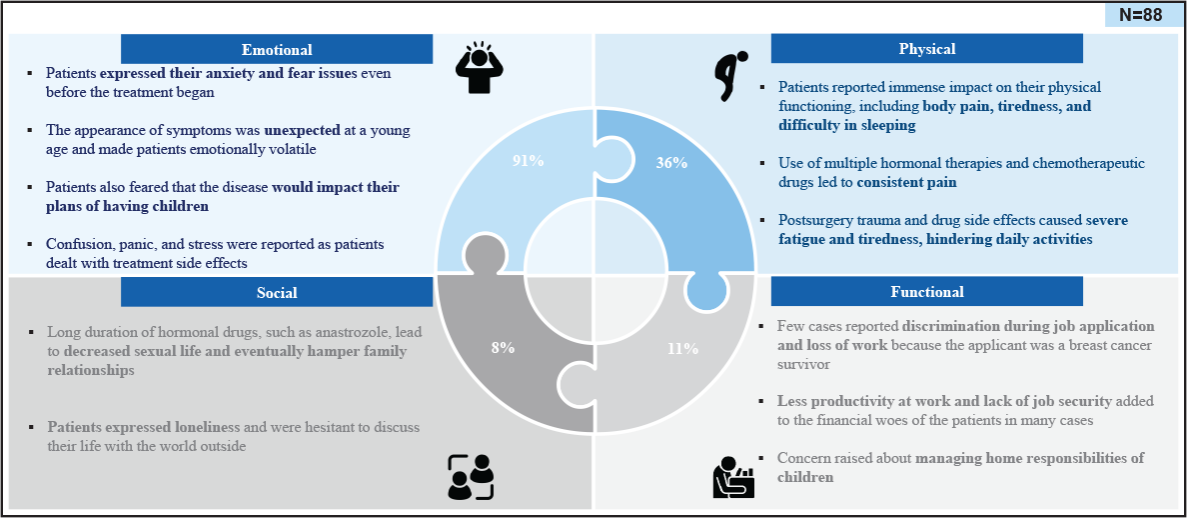
Impact of disease on quality of life of patients with early breast cancer. “N” denotes the number of posts.

A total of 88 posts included discussions regarding QoL. eBC substantially impacted patients’ QoL, affecting emotional well-being (80/88, 91%) on domains, such as anxiety and fear; physical functioning (32/88, 36%) in body pain, tiredness, and difficulty in sleeping; functional well-being (10/88, 11%), characterized by less productivity at work and lack of job security; and social well-being (7/88, 8%), including decreased sexual life, eventually hampering family relationships and resulting in loneliness (Figure 4).

### Recurrence and Associated Challenges

A total of 21 posts reported information on recurrence. Patients and caregivers expressed anger and disbelief upon recognizing symptoms of recurrence, which were often detected only after the cancer had metastasized to other organs. Symptoms, such as nodules and swollen breasts, were reported in 24% (5/21) of the posts on relapse. This led to repeated diagnostic tests, including CT, MRI, and PET scans as described in 33% (7/21) of the posts. The increase in disease activity on imaging tests was associated with feelings of devastation, as described in 33% (7/21) of the posts.

Recurrence had a profoundly negative impact on patients’ QoL, causing frustration with the repetitive diagnostic tests and the treatment process. Overall, 19% (4/21) of the posts highlighted delays in disease monitoring, often due to false positive or negative biomarker results. Instances of negligence by radiologists were mentioned, with 10% (2/21) of the posts reporting that symptoms were initially ignored, leading to a later diagnosis of recurrence after seeking second opinions. Additionally, the long waiting periods for treatment in some areas of Italy prompted some patients to consider relocating for faster access to care, as reported in 10% (2/21) of the posts.

Considering 12 posts reporting data on remission, 59% (7/12) of the posts reported that patients were in remission for more than 6 years, 33% (4/12) for 3‐6 years, and 8% (1/12) for less than 3 years.

**Figure 5.**
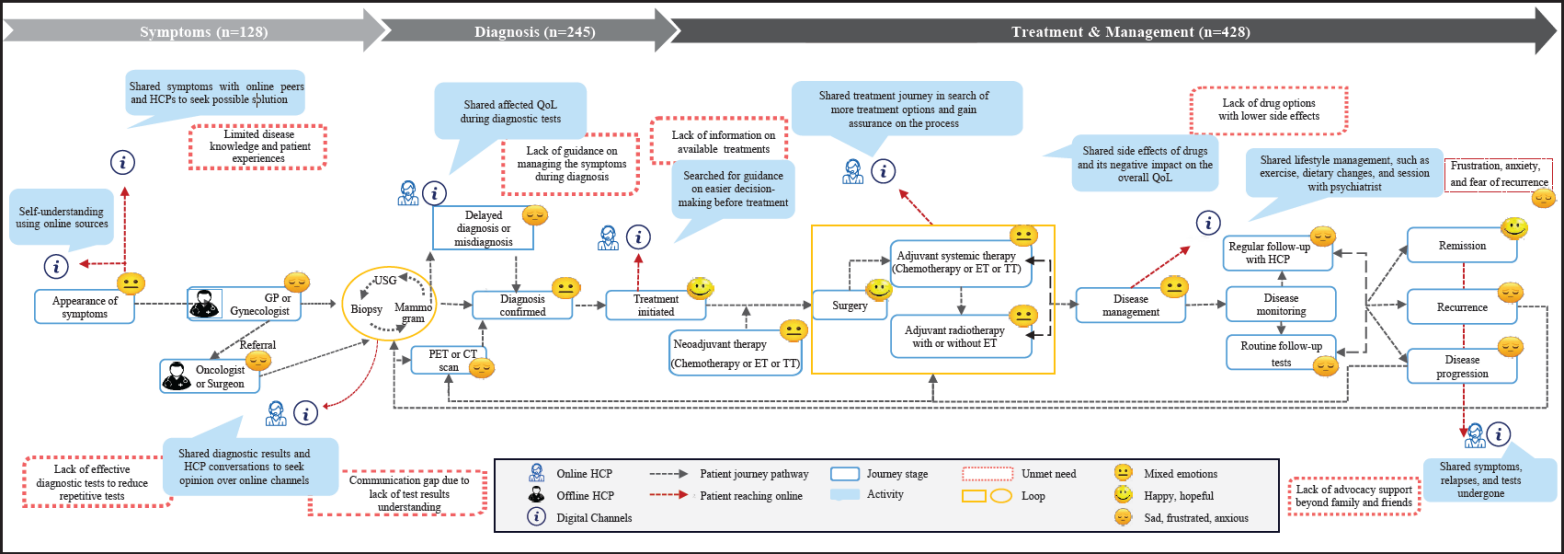
Digital touchpoints across the patient journey map. ”n” denotes the number of mentions of the specific attribute. CT: computed tomography; ET: endocrine therapy; GP: general practitioner; HCP: health care professional; MRI: magnetic resonance imaging; PET: positron emission tomography; QoL: quality of life; TT: targeted therapy; USG: ultrasonography.

The patient journey highlighted the reliance on digital channels for information, support, and communication through different stages, while it highlighted both online and offline interactions. Throughout their journey, patients experienced various emotions, including anxiety, sadness, frustration, hope, and happiness. Positive emotions were typically associated with successful treatments and management, while negative emotions often resulted from misdiagnoses, treatment side effects, and disease recurrence. Figure 5 illustrates the patient journey map, focusing on digital touchpoints—online platforms (Facebook, X, Instagram, YouTube, forums, and blogs) where patients seek information, support, and communicate with HCPs and peer networks—at various stages: symptoms, diagnosis, and treatment and management.

### Insights From HCPs

This study highlighted optimism among HCPs regarding advancements in eBC care. They shared research through book publications focusing on diagnostic techniques and provided guidance on radiation therapy. Clinical advancements were discussed, including the role of artificial intelligence in BC management, phase 3 clinical trial results, and insights on new drugs for BC. Furthermore, a lot of emphasis was placed on raising disease awareness among patients. This included discussions on proactive measures for fertility preservation, sharing advancements in screening techniques, and underlining the importance of regular check-ups. HCPs also emphasized the harmful impacts of alcohol consumption on BC risk. They actively engaged patients through various channels to promote better disease management and prevention.

**Figure 6.**
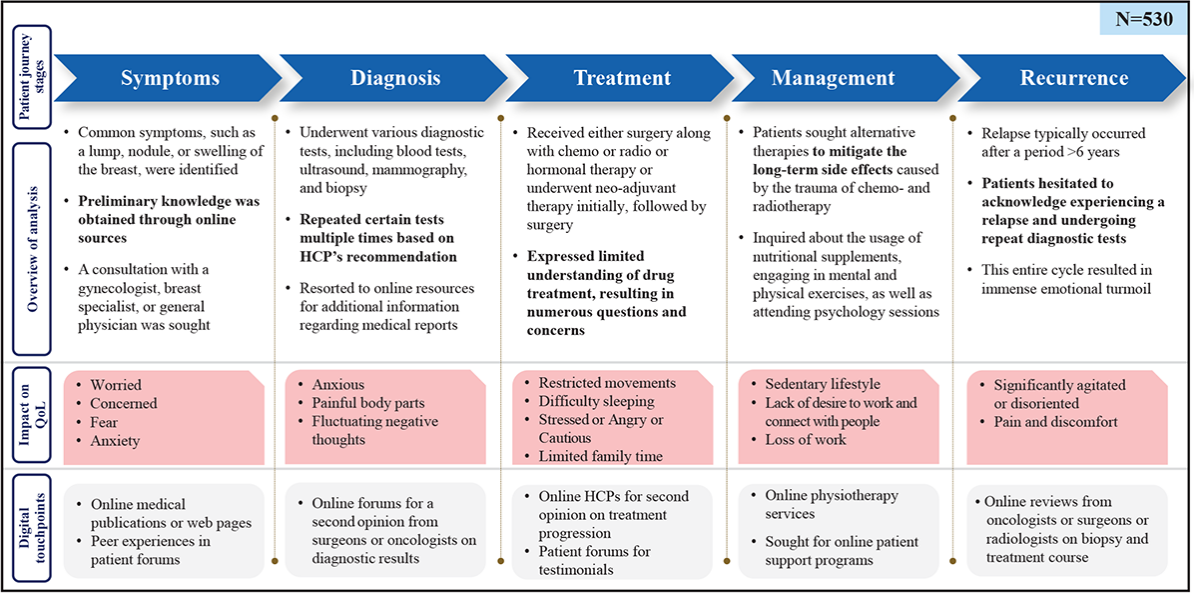
Summary of insights at various stages of the journey of patients with early breast cancer (eBC). “N” denotes the number of posts. HCP: health care professional; QoL: quality of life.

A summary of insights from this study at various stages of the journey of patients with eBC is shown in Figure 6.

## Discussion

### Overview of Findings

#### Key Themes Identified Across the Journey of Patients With eBC in Italy

This SML study provides insights into how patients with eBC, caregivers, and HCPs in Italy describe their experiences, perceptions, and unmet needs on social media. Through the analysis of posts from various social media platforms over a 2-year period, we identified recurring themes related to initial symptoms and diagnosis, treatment experiences and concerns (including adjuvant therapies), impact on QoL, perceived recurrence and ongoing monitoring, communication gaps with HCPs, and access to support and specialized care. These findings underscore the significance of social media as a supplementary resource for understanding patient experiences in real-world settings and highlight critical areas that necessitate attention to improve patient care and support systems.

#### Social Media Landscape and Online Behavior

This study reveals that most social media posts were initiated by patients themselves, highlighting their willingness to share experiences and seek peer support. This aligns with previous research that indicated that patients are increasingly turning to online platforms for emotional and informational support [16,30]. The prevalent use of forums and X emphasizes the role of these platforms in disseminating information and fostering community engagement [14,17]. However, as the sample is limited to users who are active online, these insights may reflect the perspectives of more digitally engaged patients rather than all individuals with eBC.

#### Initial Symptoms and Diagnosis

The most common symptoms reported in this study include lumps, cysts, and breast pain. These early indicators drive patients to seek diagnostic interventions, such as mammography, biopsy, and ultrasound, with patients having varied anxiety levels toward these methods.

Patients frequently discussed the diagnostic journey, expressing concerns about the multiple tests and the associated waiting periods. These findings were similar to those of a previous study that found that prolonged diagnostic processes contribute to substantial patient anxiety and stress [31]. Patients’ inability to describe their disease stage during discussions indicated a generally low level of awareness about the disease and its clinical characteristics (eg, staging and interpretation of test reports). Consistent with previous research [32,33], the findings of this study indicated that self-examination for BC symptoms, proactive communication from gynecologists or breast specialists, and clear guidance from oncologists were key drivers for diagnosis, supporting the early identification of BC and the potential for improved survival outcomes. Given the nature of SML data, these observations are hypothesis-generating and should be interpreted as qualitative insights rather than definitive epidemiologic estimates.

#### Treatment Experiences and Sentiments

Patients also expressed great concern regarding the long durations and side effects of chemotherapy and endocrine therapy, leading them to seek alternative treatment strategies. This aspect of patient experience is well documented in the literature, where adverse effects of treatment often result in diminished QoL [34]. The challenges in understanding the complexities of adjuvant therapies indicate a need for more transparent communication from HCPs [4]. Furthermore, negative perceptions of adjuvant therapies significantly impact treatment adherence. Prolonged low adherence or nonadherence increases the risk of recurrence, ultimately leading to poorer patient outcomes [35-37]. In our dataset, patients often described doubts about the benefits of ongoing treatment and concerns about side effects. Although actual adherence behavior could not be measured, these narratives signal areas where enhanced patient-HCP dialogue and shared decision-making may be beneficial.

The discussions reflected a lack of open communication from HCPs regarding treatment options. Similar findings were observed in a social media study conducted on patients with metastatic BC from Europe [19]. Collectively, these findings indicate that enhancing the clarity, frequency, and empathy of patient-HCP communication may help address some of the concerns expressed in online discussions.

#### Impact on QoL

The impact of eBC on patients’ QoL was another critical theme identified in this study. Consistent with the literature, the findings of this study show that eBC substantially affects emotional well-being, physical functioning, functional well-being, and social well-being [38]. Patients reported anxiety, fear, pain, and fatigue, which also influenced their productivity at work and personal relationships. The prevalence of emotional distress among patients with eBC underscores the importance of comprehensive supportive care that addresses both the physical and psychological needs of patients [34,39-42]. Because the data were derived from spontaneous posts, individuals experiencing higher levels of distress may have been more likely to share their experiences online, potentially amplifying the prominence of negative QoL themes.

#### Recurrence, Ongoing Screening, and Continuous Therapy

The findings of this study indicated that recurrence had a substantial impact on QoL, necessitating regular imaging tests and repeated cycles of chemotherapy, which became an integral part of the treatment journey. This study highlights a critical unmet need for timely and accurate monitoring to prevent recurrence and mitigate long-term impacts. Although we could not independently verify clinical details, such as recurrence status or specific testing protocols, repeated patient discussions regarding continuous imaging, biomarker testing, and changes in therapy underscore the burden of long-term surveillance from the patient perspective.

#### Unmet Needs and Communication Gaps

Despite the availability of various treatment options, a notable number of patients indicated unmet needs in treatment knowledge and patient-HCP communication. The lack of trusted online peer groups and limited access to specialty breast care units were also prominent concerns. These findings are supported by previous studies [43-45] that emphasized the importance of accessible, timely, and accurate information for patients to make informed decisions about their care, as well as the role of patient advocacy groups [46].

Poor patient-HCP communication was frequently mentioned, with patients expressing dissatisfaction with the clarity and frequency of consultations. Patients complained about multiple tests and ineffective communication with HCPs about test results, prompting patients to seek help online. Effective communication is critical in oncology care, yet often remains suboptimal, contributing to patient distress and a sense of being unsupported [11,19]. This suggests that targeted interventions to improve patient-HCP interactions could enhance patient satisfaction and perceived outcomes.

#### Role of HCPs and Social Media

HCPs also engaged actively on social media, sharing advancements in eBC care and offering guidance on disease management. This active participation aligns with the findings from previous studies that underscore the role of social media in bridging knowledge gaps and fostering public health education [47,48]. The discussions by HCPs on clinical trials, diagnostic techniques, and preventive measures, such as regular screenings and lifestyle modifications, reflect an ongoing effort to raise disease awareness and improve patient care. Nevertheless, our analysis suggests that HCP-driven content may not always directly address the specific questions and concerns raised by patients and caregivers, indicating an opportunity to better align online communication with patient or caregiver needs.

#### New Insights From the Findings of This Study

New insights from the findings of this study are as follows:

1. This SML study revealed gaps that are not typically captured by traditional research methods, including the generational shift in how younger patients (aged 31‐40 years) are using online platforms as their primary source of both emotional and informational support. This highlights an urgent need for formal integration of digital tools into cancer care frameworks, for example, through moderated online communities, validated educational content, and digital navigation tools.
2. The study provides new evidence regarding the reported geographic inequities in access to care. Patients in underserved areas reported delays in treatment and follow-up, highlighting a systemic issue that could be addressed with regional policy innovations, such as expanding specialized breast care units or creating mobile health care services. These insights should be interpreted cautiously as they are based on self-reported locations and experiences, but they point to priority areas for further health services research.
3. Recurring diagnostic inaccuracies, particularly in biomarker testing during recurrence monitoring, emerged as a unique and actionable finding. While specific clinical details could not be confirmed, addressing these perceived inaccuracies through standardized protocols and advanced imaging techniques could significantly alleviate patient distress and potentially improve outcomes. Future studies combining SML with clinical data or registry information may help validate and quantify the extent of these issues.

### Strengths and Limitations

This study has several strengths and limitations. One key strength is its relatively novel approach, utilizing SML to gather real-world insights into patient experiences and perceptions without intruding on their privacy. This method captures spontaneous and candid discussions among patients, caregivers, and HCPs that are difficult or time-consuming to elicit through traditional research methods, such as surveys or interviews. A second strength is the breadth and diversity of data sources. Posts were collected from multiple social media platforms (X, Facebook, forums, blogs, Instagram, and YouTube), which provides diverse perspectives and a broader understanding of the journey of patients with eBC in Italy. Third, the study benefits from a systematic methodological framework. The search strategy was developed by a multidisciplinary team and iteratively refined with scoping searches and clearly defined eligibility criteria. Data processing combined NLP-based filtering and manual review to exclude junk, spam, and irrelevant content and focus on posts reflecting experiences of patients, caregivers, and HCPs. Thematic analysis was guided by a patient-journey–based conceptual framework. Together, these elements enhance the methodological rigor, transparency, and reproducibility of the study. Finally, the study generated a substantial volume of qualitative material for a focused national context. From 20,008 initially extracted posts, 530 relevant posts were retained for detailed analysis, enabling an in-depth exploration of specific themes, such as diagnostic pathways, perceptions of adjuvant therapies, QoL impacts, recurrence-related challenges, communication gaps, and access barriers.

However, the study has certain limitations. First, the social media users included in the analysis may not be representative of the broader population of patients with eBC, resulting in a potentially nonrepresentative sample. We attempted to mitigate this by including multiple platforms and both Italian- and English-language posts from users in Italy. However, selection bias toward more digitally engaged, younger, or health-literate individuals is likely. This may overemphasize certain themes (eg, online information-seeking) and underrepresent less connected or older populations. Future studies could integrate SML with survey or registry data to improve generalizability. Second, as the study relies on self-reported information, the accuracy and completeness of the data may vary, and it is not possible to verify the authenticity of individual posts. Third, the retrospective, cross-sectional design captures content from a specific period, from December 2021 to November 2023, which may not fully reflect changes over time or emerging trends. As such, temporal shifts in treatment paradigms, public awareness, or health policy may not be fully captured. Longitudinal SML analyses or repeated cross-sectional assessments could provide a more dynamic picture of evolving patient needs. In addition, the anonymous nature of social media data from publicly available posts prevents tracking individual users across platforms and precludes the verification of key demographics or clinical characteristics (eg, age, disease stage). This limits the ability to draw robust conclusions about specific subgroups. Our subgroup insights (eg, younger patients or those in underserved regions) should therefore be interpreted as indicative patterns rather than statistically confirmed differences. Future work that links deidentified social media data with structured demographic or clinical information, where ethically and legally permissible, could refine these subgroup analyses. Finally, the quality, depth, and specificity of shared information differ considerably between the posts, which may introduce gaps and uncertainty in data analysis and interpretation.

These strengths and limitations provide a comprehensive understanding of the study and emphasize the need for additional research and ongoing methodological improvements in the use of SML to inform patient-centered cancer care.

### Conclusions

This study highlights the utility of SML in understanding the nuanced experiences of patients with eBC, caregivers, and HCPs in Italy, as expressed through publicly available social media posts. The identified themes reveal critical unmet needs in patient education, support systems, access to specialty care units, and patient-HCP communication. While the findings are exploratory and reflect the perspectives of social media users rather than all patients with eBC, they provide valuable, real-world insights that can complement traditional research approaches. Addressing these gaps through targeted interventions and leveraging social media’s potential for tailored information, peer support, and enhanced patient-HCP communication can enhance patient experiences and perceived outcomes. Further research is encouraged to develop and implement effective strategies for improving cancer care in the digital age, including the integration of digital health tools, evaluation of patient-HCP communication interventions, and linkage of SML data with clinical and health services datasets where feasible and ethical.

## Supplementary material

10.2196/73371 Multimedia Appendix 1 Search terms, inclusion and exclusion criteria, and list of forums and blogs.

10.2196/73371 Multimedia Appendix 2 Demographic and clinical characteristics.

10.2196/73371 Multimedia Appendix 3 Insights from various social media channels.

10.2196/73371 Checklist 1 SRQR checklist.

## Abbreviations

BC: breast cancer
CT: computed tomography
eBC: early breast cancer
HCP: health care professional
HIPAA: Health Insurance Portability and Accountability Act
MRI: magnetic resonance imaging
NLP: natural language processing
PET: positron emission tomography
QoL: quality of life
SML: social media listening
SRQR: Standards for Reporting Qualitative Research
