# Supplementary material for: Exploring the Experiences and Perspectives of Patients With Early Breast Cancer, Caregivers, and Health Care Professionals: Italian Social Media Listening Study
Source: JMIR Cancer. 2026 Mar 24;12:e73371. doi: 10.2196/73371 (PMC13012224; doi:10.2196/73371)
Supplement: Multimedia Appendix 2 [file cancer-v12-e73371-s002.docx]

Multimedia Appendix 2. Demographic and clinical characteristics.

| **Characteristic** | **Percentage (n/N)** |
| --- | --- |
| **Stakeholder segmentation** |  |
| Patients | 60 (318/530) |
| Caregivers | 21 (111/530) |
| HCPs | 16 (85/530) |
| Unidentified persons | 3 (16/530) |
| **Cancer type** |  |
| De Novo | 95 (427/448) |
| Recurrent | 5 (21/448) |
| **Patient’s current age** |  |
| 21-30 years | 13 (12/89) |
| 31-40 years | 26 (23/89) |
| 41-50 years | 24 (21/89) |
| 51-60 years | 13 (12/89) |
| 61-70 years | 16 (14/89) |
| >70 years | 8 (7/89) |
| **Time since diagnosis** |  |
| 6 months to 1 year | 31 (28/90) |
| 1-3 years | 21 (19/90) |
| 4-6 years | 12 (11/90) |
| > 6 years | 36 (32/90) |
| **Caregiver relationship with patient** |  |
| Child | 53 (57/107) |
| Friend | 12 (13/107) |
| Spouse | 10 (11/107) |
| Sibling | 10 (11/107) |
| Family | 6 (6/107) |
| Grandchild | 6 (6/107) |
| Mother | 3 (3/107) |

HCPs: health care professionals
