# Supplementary material for: Exploring the Experiences and Perspectives of Patients With Early Breast Cancer, Caregivers, and Health Care Professionals: Italian Social Media Listening Study
Source: JMIR Cancer. 2026 Mar 24;12:e73371. doi: 10.2196/73371 (PMC13012224; doi:10.2196/73371)
Supplement: Multimedia Appendix 3 [file cancer-v12-e73371-s003.pdf]

N=530

## Forums & Blogs (49%)

- Patients used online forums to discuss **coping with drug side effects** and **the impact of their disease on well-being**
- The forums **'Medicitalia'** and **'Carenitiy'** were preferred by patients due to the **active response from HCPs**
- **HCPs hosted their own blog pages** to share new advancements, including AI detecting breast cancer
- The blogs served to provide **accurate information** and **prevent treatment misinformation**

## Twitter/X (31%)

- HCPs predominantly used Twitter/X to raise disease awareness by highlighting **risk factors like smoking and alcohol consumption**
- HCPs also engaged in discussions about **recent clinical trials** and **upcoming advancements** related to topics such as **"Irradiation and Breast conserving surgery"** and **"pausing therapy for conceiving and its associated risk of recurrence"**
- Patients used Twitter/X to share their personal stories and **encourage their peers to make mammography** a routine practice

## Facebook (13%)

- Community pages like **'Tumore al seno'** were rich in patient conversations where individuals **sought advice** from their peers and **shared their experiences** in managing the disease
- Patients were **comfortable discussing their concerns regarding bodily changes,** such as breast appearance or nipple discharge
- Patients shared their stories through various modes, including **text, pictures, and videos**

## Instagram/ YouTube (7%)

- Instagram served as a platform for raising awareness through the following methods:
- Providing **coverage of congress events,** such as the ESMO session on the impact of adjuvant ribociclib on HR-QoL in eBC
- Conducting **interviews with experts** on various topics, including the mental aspects for patients with eBC
- **Local patient support groups shared testimonials from patients on YouTube**, shedding light on issues like delayed diagnosis and undergoing multiple tests

***Prominent reporter type***

Patient | Caregiver | HCP | Patient support group/Online peer
