# Supplementary material for: Exploring the Experiences and Perspectives of Patients With Early Breast Cancer, Caregivers, and Health Care Professionals: Italian Social Media Listening Study
Source: JMIR Cancer. 2026 Mar 24;12:e73371. doi: 10.2196/73371 (PMC13012224; doi:10.2196/73371)
Supplement: Checklist 1 [file cancer-v12-e73371-s004.docx]

Multimedia Appendix 1. Standards for Reporting Qualitative Research (SRQR) Checklist.

| **Index** | **Topic** | Page(s) |
| --- | --- | --- |
| **Title and Abstract** | | |
| 1 | Title | 1 |
| 2 | Abstract | 2-3 |
| **Introduction** | | |
| 3 | Problem formulation | 5 |
| 4 | Purpose or research question | 5 |
| **Methods** | | |
| 5 | Qualitative approach and research paradigm | 5 |
| 6 | Researcher characteristics and reflexivity | 8 |
| 7 | Context | 4 |
| 8 | Sampling strategy | - |
| 9 | Ethical issues pertaining to human subjects | 9 |
| 10 | Data collection methods | 7 |
| 11 | Data collection instruments and technologies | 7 |
| 12 | Units of study | 7-9 |
| 13 | Data processing | 7-8 |
| 14 | Data analysis | 7-9 |
| 15 | Techniques to enhance trustworthiness | 7-9 |
| **Results** | | |
| 16 | Synthesis and interpretation | 10-19 |
| 17 | Links to empirical data | 10-19 |
| **Discussion** | | |
| 18 | Integration with prior work, implications, transferability, and contribution(s) to the field. | 20-25 |
| 19 | Limitations | 25-26 |
| **Other** | | |
| 20 | Conflicts of interest | 28 |
| 21 | Funding | 27 |

**Reference:**

O’Brien BC, Harris IB, Beckman TJ, et al. Standards for reporting qualitative research: A synthesis of recommendations. *Acad Med.* 2014;89:1245–51.
